# The Cost of Universal Health Care in India: A Model Based Estimate

**DOI:** 10.1371/journal.pone.0030362

**Published:** 2012-01-27

**Authors:** Shankar Prinja, Pankaj Bahuguna, Andrew D. Pinto, Atul Sharma, Gursimer Bharaj, Vishal Kumar, Jaya Prasad Tripathy, Manmeet Kaur, Rajesh Kumar

**Affiliations:** 1 School of Public Health, Post Graduate Institute of Medical Education and Research, Chandigarh, India; 2 Department of Family and Community Medicine, St. Michael's Hospital, Toronto, Canada; 3 Centre for Research on Inner City Health, Keenan Research Centre, Li Ka Shing Knowledge Institute, St. Michael's Hospital, Toronto, Canada; Erasmus University Rotterdam, Netherlands

## Abstract

**Introduction:**

As high out-of-pocket healthcare expenses pose heavy financial burden on the families, Government of India is considering a variety of financing and delivery options to universalize health care services. Hence, an estimate of the cost of delivering universal health care services is needed.

**Methods:**

We developed a model to estimate recurrent and annual costs for providing health services through a mix of public and private providers in Chandigarh located in northern India. Necessary health services required to deliver good quality care were defined by the Indian Public Health Standards. National Sample Survey data was utilized to estimate disease burden. In addition, morbidity and treatment data was collected from two secondary and two tertiary care hospitals. The unit cost of treatment was estimated from the published literature. For diseases where data on treatment cost was not available, we collected data on standard treatment protocols and cost of care from local health providers.

**Results:**

We estimate that the cost of universal health care delivery through the existing mix of public and private health institutions would be INR 1713 (USD 38, 95%CI USD 18–73) per person per annum in India. This cost would be 24% higher, if branded drugs are used. Extrapolation of these costs to entire country indicates that Indian government needs to spend 3.8% (2.1%–6.8%) of the GDP for universalizing health care services.

**Conclusion:**

The cost of universal health care delivered through a combination of public and private providers is estimated to be INR 1713 per capita per year in India. Important issues such as delivery strategy for ensuring quality, reducing inequities in access, and managing the growth of health care demand need be explored.

## Introduction

In the midst of a massive global financial crisis, India's economy continued to grow. It can boast of a growing middle class and advanced technological developments in the industries [1]. It is the world's largest democracy, with an independent media and strong civil society. However, India is ranked 119 out of 169 countries in terms of human development index (HDI) [2]. When adjusted for increasing inequality, the value of HDI falls by 32% for India [3]. The enormous growth in wealth has not been distributed equally and the gap between the health of the richest and poorest continues to widen in India [4]. The coverage of immunization, which is a service provided free of cost in public sector facilities, is nearly 3 times among children belonging to highest income quintile households (71%) as compared to poorest quintile children (24%) [5]. An important reason for such disparities is inequitable access to health services. The poor and middle classes do not have the same level of access to high quality health care as the wealthy [4], [5], [6], [7], [8].

Inequitable access to healthcare occurs due to a myriad of factors, but is rooted in a low overall financing of healthcare by the State. India spends only 5% annual gross domestic product (GDP) on health care [9]. Of this, most of the expenditure (about 80%) is private out-of-pocket. High out-of-pocket costs make health services inaccessible to a significant proportion of Indian households. Among those who decided not seek medical care for an ailment, nearly 20% of urban and 28% rural households cited financial constraints as the limiting factor [10]. Among many of those who had to purchase health care, out-of-pocket expenditures prove catastrophic. In India, nearly 3.1 million additional households slip to levels below the poverty line ($1 per day) per annum as a result of hospitalization expenditure [11].

Those accessing health care in the public sector generally receive poor quality services. Previous studies from India have cited reasons such as high absenteeism, poor quality of services, rampant corruption and long travel distances as prominent reasons for poor access of public sector health facilities [12], [13], [14], [15]. Those accessing private providers largely encounter unlicensed practitioners who deliver poor quality care. High costs exist in private sector because of a lack of regulation. Market liberalization has predictably led to flourishing of private market. One area that has been greatly impacted by liberalization has been the cost of medicines. Reduction in the number of drugs under price control has lead to a 197% increase in prices of 778 drugs in a period between 1980 to 1995 [16].

Given this situation, there have been a number of calls to achieve universal access to health care services. A Task Force has been commissioned in 2010 by the Government of India, to develop a framework for universal health care, as part of the 12^th^ Five Year Plan. One of the strategies put forth by the Task Force includes creation of one single pool with options to purchase services from public and private providers [17]. The National Health Bill which is currently under consideration aims to “provide for protection and fulfillment of rights in relation to health and well being, health equity and justice” [18]. Such initiatives call for a basic package of health services to be available to all citizens. There is an urgent need to explore important questions such as how much would this package of services cost, how it would be financed, and how it would be delivered.

World Health Organization estimates that for providing universal health care public sector health care outlays should be increased to approximately 6% of GDP in low income countries [19]. Reddy et al (2011) also suggest similar estimates for providing health care universally in India, although their methodology is not comprehensively described [17]. National Macroeconomic and Health Commission had estimated the healthcare expenditure of INR 1160 per capita [20]. Using three different cost estimates assumptions from Thailand, Mexico and India and making adjustments for income differences and inflation, Mahal et al (2011) estimate for providing health care to 90% population ranged from less than 2% of GDP (with Mexico costs) to greater than 4.3% (with Thailand costs) [21]. In this study, we estimate the cost of providing basic package of health care services as outlined in the Indian Public Health Standards (IPHS), based on the costs in Chandigarh, a large city in northern India. We use the model to estimate the cost of universalizing health care in India. Lastly, we discuss the feasibility of delivering universal health care through public and private providers, as proposed by the Task Force on Universal Health Care (Table 1).

**Table 1:** Feasibility* of universalizing health care using a model of public and private sector delivery in India.

	Feasibility Criteria	Current Context/Proposed mechanism for provision of universal health care in India	Conclusion	Source
Consumer:				
	Willing to pay premium	Sizable population in India lacks willingness to pay and thus Government needs to commit the cost of universal health care.	Favourable	
Government:				
	Willing to involve private sector in delivery of health care.	Government has shown increasing commitment and willingness to involve private sector in delivery of health care through different schemes.	Favourable	[48]
	Willing to administer hospital autonomy.	Through establishment of *Rogi Kalyan Samitis* (RKS), Government has devolved some administrative and financial powers to autonomous institutional bodies.	Favourable	[48]
	Willing to charge higher user fees in hospitals.	Fixing user fees at optimal levels (to be paid by government), will create revenues for public sector hospitals which can then be used to improve quality of services by incentives and compete with private sector.	Favourable	
	Ability to manage funds.	Insurer-manager models have been successfully tried (in RSBY, Aarogyashri and ESIS), however will require further strengthening for creating a national health fund.	Requires strengthening	
	Ensure mandatory population participation to avoid adverse selection.	Our proposed model requires coverage of an entire geographic population hence it minimizes adverse selection possibility.	Favourable	[43]
	Estimation of cost of care.	The present study estimates cost of care. These estimates can be further refined by better availability of cost and disease burden data and through yardstick competition principle.	Requires further strengthening	
Private Sector:				
	Profitable for private sector participation.	The current estimates of cost and payments have taken into account the salary structures of private sector, and provision of care with standard protocols; and hence should be acceptable to private sector.	Favourable	
Organizations for fund management:				
	Capacity development.	Will require strengthening for monitoring capacity and establishment of supportive legislations for smooth implementation	Requires strengthening	

*Feasibility evaluation matrix adapted from Hotchkiss D et al (1999) [49].

## Methods

### Setting

Chandigarh is a city of over 1,000,000 people in northern India. It serves as the capital of two adjacent states, Punjab and Haryana. A range of public and private health care institutions provide preventive and curative health care. Public sector institutions include 2 tertiary care and 2 secondary care hospitals, 3 community health centers and 26 dispensaries. Over 80 private clinics, nursing homes and hospitals constitute the private sector, which mostly provides secondary care, with few providing tertiary care services.

### Model

**Figure 1:**
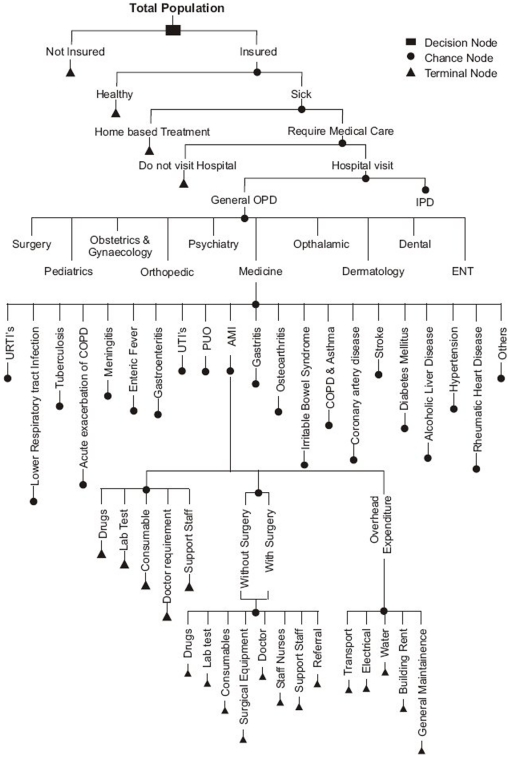
Decision Model to predict patient load, human resource and recurrent cost to a hospital.

We collected data to parameterize a decision model to estimate the cost associated with delivering health care to a population of 100,000 people in Chandigarh (Figure 1). Later we use the variation in burden of disease, treatment seeking behavior, cost of care and other model parameters to generalize our study results to India, and to assess the fiscal implications of implementing universal health care.

#### Benefit Package

We outlined a package of services as using Indian Public Health Standards (IPHS) [22]. The overall objective of these IPHS standards has been to define a comprehensive package of quality services, responsive to the needs of the community. The services to be included at the district hospitals include general and specialist out-patient department (OPD), in-patient department (IPD) and emergency services; laboratory and diagnostic services; preventive services and; other administrative and support services. These services were applicable to serve all sections, and were defined after stratification of hospitals according to number of beds, i.e. 100, 200, 300, 400 and 500 [22].

#### Disease Burden Estimation

Morbidity rates were derived from National Sample Survey (NSS) data (2004–05) for India to estimate the probability of falling ill and reporting to hospital to receive medical care on a monthly basis [10]. Morbidity rate derived from NSS data was increased by a factor of 1.5, to develop a conservative estimate of patient need. This incremental factor was applied considering that there would be increased demand for services following a reform, whereby no price is attached to service delivery (Text S1). NSS data from 60^th^ round (2004–05) shows that about 30% of those who did not access curative service following an illness, cite financial reasons for not seeking treatment for a given episode of illness [6]. We believe that this 30% of non-users would also start using treatment services.

Service data for one year (2008–09) from two tertiary and two secondary care hospitals in Chandigarh was used to derive proportional utilization rates of OPD & IPD and proportional morbidity rates among each specialty (Text S1). This administrative data was thus used to develop a morbidity profile for the community. Additionally, we used this data to derive patterns of treatment (e.g. in-patient care vs. out-patient care) and average length of stay (Table 2). All four of these hospitals are publicly funded and publicly administered. Given that they cater to the majority of demand for curative services, they are fairly representative of the morbidity profile of the population. We estimated specialty-wise and disease-wise distribution of OPD and IPD attendance (Text S1).

**Table 2:** Model Parameters and Sensitivity Analysis for Annual Household Costs for provision of health care to 100,000 population.

				Annual Cost per Household (INR)			
		Uncertainty range		Using Branded Drugs		Using Generic Drugs	
Parameter	Base value	Lower	Upper	Lower	Upper	Lower	Upper
Total Population	100000	80000	120000	7900	8361	6355	6816
Morbidity rate	0.1	0.08	0.12	6695	9474	5459	7619
Adjusted Proportion of patients reporting to hospital	1.2	1.125	1.425	7650	9387	6202	7552
Monthly patient load reporting to hospital	12000	7200	41040				
Cost ratio (IPD:OPD)	10	8	15	7999	8655	6444	6924
Proportion Patients treated (IPD basis)	0.05	0.02	0.1	7875	8433	6330	6888
Correction factor (patients treated on IPD basis)	0.3	0.2	0.5	7899	8456	6358	6901
Number of OPD days in a month	24	24	24				
Average no. of individuals per family	4						
**Distribution OPD patients (Proportional Morbidity Rates)**							
General medicine	0.22	0.12	0.32	6898	9218	5803	7243
Oto-rhino-laryngology	0.07	0.01	0.11	7810	8427	6155	6818
Ophthalmology	0.07	0.01	0.11	7380	8609	5874	7034
Dermatology	0.12	0.06	0.16	8026	8296	6454	6669
Gynecology	0.14	0.04	0.24	7492	8846	6022	7113
Psychiatry	0.02	0.005	0.025	8104	8259	6521	6636
Pediatrics	0.12	0.06	0.16	7779	8315	6290	6728
Surgery	0.11	0.05	0.15	8000	8330	6396	6726
Dental	0.05	0.01	0.09	8013	8349	6426	6727
Orthopedics	0.09	0.04	0.14	8020	8352	6415	6747
**Specialty-wise average length of IPD stay (days)**							
OBG	4.5	3	7	8159	8234	6554	6629
Eye	2.6	1	5	8168	8215	6563	6610
Surgery	5.6	3	9	8129	8262	6524	6657
Medicine	6.9	4	10	8131	8247	6526	6642
ENT	3.7	2	5	8169	8199	6564	6595
Psychiatry	16.2	6	26	8182	8190	6577	6585
Pediatrics	6.9	4	10	8145	8231	6540	6726
Orthopedics	10	7	20	8129	8374	6524	6769
Skin	6.3	3	10	8152	8225	6547	6621

#### Standard Treatment Protocols

Standard treatment protocols were used to estimate the cost of treating common diseases that represent 90% of the total disease burden in each specialty. Some of these protocols were developed from an existing Government of India and World Health Organization study done in 2007 [20]. For the remainder diseases which constituted about 44% of total disease burden, we consulted the experts from respective departments of a public sector tertiary care hospital on standard treatment protocols. We analyzed one year (2008–09) service data from four public hospitals in Chandigarh to estimate volume of patient load in OPD and IPD, patient bed-days and requirement of patient beds.

#### Cost of Services

The cost of treatment for select conditions was obtained from a previous consultative group report [20]. For diseases which were not covered in this report, we computed the cost of providing treatment, by estimating the cost of administering standard treatment protocols. For estimating these costs, unit cost of medicines (branded and generic), consumables and diagnostic tests was obtained from the rate contract prices available from a tertiary care public sector hospital in Chandigarh, India [23]. This hospital price was used as it is assumed that a hospital which provides care at such a scale would be able to exercise geographic monopsony in obtaining drugs and other input resources at less than the prevalent market prices. Average unit price of items, which were not available in the rate contract list of this tertiary care hospital, were obtained from a survey of three medical and surgical distributors, which had the greatest volume of sales in the city. The cost of hospitalized treatment was assumed to be 10 (8–15) times the OPD care. This assumption was based on the estimates of cost of outpatient consultation and inpatient treatment for South East Asian countries, produced by the WHO-CHOICE study [24]. For preventive services, we used a cost of INR 300 per capita per year which was estimated by the National Commission on Macroeconomics and Health report for India [25].

The cost of surgeries was obtained from costing of surgeries done by Haryana Health Department for the Surgery Package Scheme [26]. This was scaled down by 50% since this was the actual cost incurred by hospitals on respective surgeries [26]. Cost of care was reduced by a factor of 50%, based on consultation with the experts who had estimated the package rates for Haryana Government as to make it representative of the actual price of care to the hospital.

Human resource requirement has been estimated based on the predicted quantum of work, and number of hours of work available per day with each category of staff. Time available for work as in the previous report was used in our analysis [20]. This was further validated with IPHS norms. Current salary structure of different staff members obtained from a survey of four private hospitals in Chandigarh - which had maximum patient load - was multiplied with the number of staff members to obtain total salary expenditure for the hospital. Lack of adequate monetary incentives has been cited as one of the major reason for supply side shortfall in public sector [12], [13], [15]. So we collected data from private sector hospitals alone, in order to estimate cost of human resources, as we believe that in order to retain and induce performance from health workforce, it is important to pay equivalent of market prices (Text S1).

### Sensitivity Analysis

In order to generalize our results to other cities and regions in India, we varied our disease burden and treatment seeking behavior estimates to adjust for the state-wise variation in India [6]. For parameters on which state-specific information was not available, we varied the base value by 20% on either side. The 20% variation was based on the variation in morbidity rate of different states from the all-India average. Using these limits, we did a univariate sensitivity analysis to elicit the effect of each parameter on the overall annual premium value at individual and household level and the health sector budgetary outlay as a percentage of gross domestic product (GDP) (Figure S1). Secondly, we undertook a probabilistic sensitivity analysis to simulate our annual household and individual premium 1000 times. The distribution of simulated premium values was used to compute 2.5^th^ and 97.5^th^ percentiles for premium and overall cost to Government of India as a percent of GDP.

## Results

### Services

We estimated that in order to treat patients on OPD basis to serve a population of 100,000, the hospital needs to recruit four specialists in the department of Medicine, two specialists each in surgery, gynecology, pediatrics, anesthesiology and skin, and one specialist each in ophthalmology, oto-rhinolaryngology (ENT), orthopedics, psychiatry and pathology. Thirty eight medical officers and 64 staff nurses will be required for providing care on in-patient basis and in emergency department. Among other staff, 10 technicians, 29 attendants and 23 other support staff will be required. We estimated requirement for a total of 20 auxiliary nurse midwives (ANM) for providing home visits. An eleven member team will be needed for hospital administration and management.

Specialty-wise contribution of patient-visits and bed-days is shown in table 3. In order to provide medical care to a population of 100,000 individuals, we estimate that 66 beds will be required for in-patient treatment (Table 3).

**Table 3:** Specialty-wise disease burden and recurrent cost (diagnostic and medicines) incurred to hospital for treatment on OPD and IPD basis.

			Recurrent Monthly Treatment Expenditure using Branded Drugs			Recurrent Monthly Treatment Expenditure using Generic Drugs			
Department	OPD patients visits (per month)	IPD Bed-Days	OPD	IPD	Total	OPD	IPD	Total	Relative Contribution (%)
ENT	735 (6.8)	97 (8)	463360	107519	570879	272963	59386	332349	4
Dental	554 (5.1)	2 (0.2)	346745	3658	350402	300278	3658	303936	3.6
Skin	1290 (11.9)	170 (8.3)	292776	64107	356882	168410	51121	219531	2.6
Ophthalmology	727 (6.7)	78 (9.3)	951991	588683	1540674	867041	568222	1435262	17.2
Psychiatry	168 (1.6)	17 (0.3)	200055	14338	214393	141003	9302	150305	1.8
Medicine	2400 (22.2)	343 (15.3)	4119882	716528	4836410	2332485	413427	2745912	33
Orthopaedics	974 (9)	488 (14.9)							0.0*
OBG	1540 (14.3)	214 (14.7)	1132900	293062	1425962	812763	221684	1034447	12.4
Paediatrics	1263 (11.7)	253 (11.3)	771727	164589	936316	566264	100540	666804	8
Surgery	1148 (10.6)	321 (17.6)							0.0*
**Sub-Total**	10800	1984	8279436	1952484	10231920	5461208	1427340	6888547	82.7
Surgical Costs					1441994			1441994	17.3
**Total**			8279436	1952484	11673914	5461208	1427340	8330541	100

*Contributions of orthopaedics and surgery are clubbed in surgical costs.

### Costs of Health Services

We estimated the total monthly cost for staff salaries to be INR 5.3 million. Overall cost of managing patients (both OPD and IPD) is 1.4 times higher when branded medicines (INR 11.6 million) were used, as compared to generic medicines (INR 8.3 million). In order to provide health services to 100000 population, annual cost per household was estimated to be INR 6,852 (INR 3,226–INR 13,099) with generic drug use (Table 4). Under the scenario of generic medicine use, this cost was found to be maximally sensitive to the overall morbidity rate, incremental factor for morbidity rate and proportional OPD attendance rates in Medicine, Ophthalmology and Obstetrics and Gynecology (Table 2, Figure S1).

**Table 4:** Annual Cost and Health Sector Budgetary Allocation for Universalizing Health Care in India.

	Cost of Care with Branded Drug Use			Cost of Care with Generic Drug Use		
Cost of Curative Care	Base	Lower Bound	Upper Bound	Base	Lower Bound	Upper Bound
Annual Cost per Person INR (USD)	2198 (49)	986 (22)	4261 (95)	1713 (38)	807 (18)	3275 (73)
Annual Cost per Household, INR (USD)	8793 (195)	3946 (88)	17044 (379)	6852 (152)	3226 (72)	13099 (291)
% GDP allocation to health (preventive and curative)	4.7	2.4	8.6	3.8	2.1	6.8

**Figure 2:**
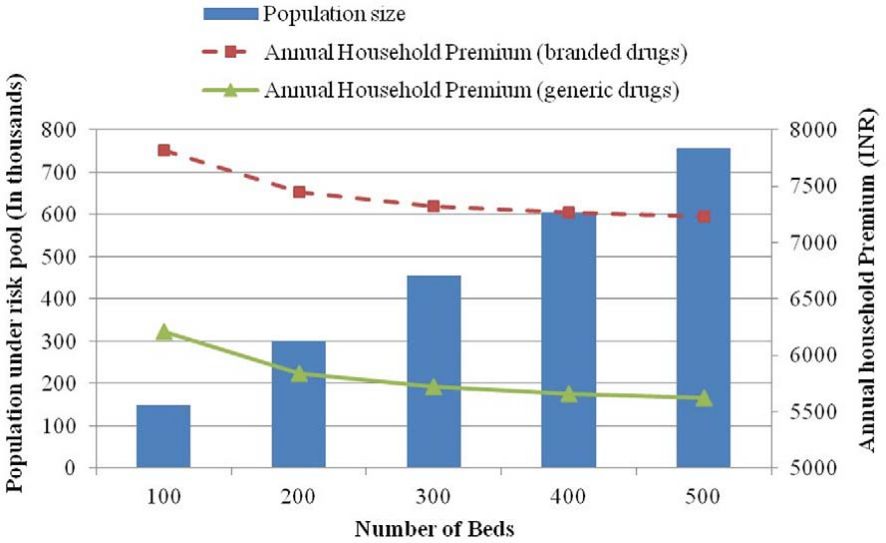
Efficient population and annual household costs for district hospitals under Indian Public Health Standards, by bed strength, India.

For hospitals to provide care, the efficient scale of population which should be covered by hospitals with 100, 200, 300 and 400 bed hospitals would be 0.15, 0.3, 0.45 and 0.6 million respectively. Similarly the annual cost per household for hospitals with 100, 200, 300, 400 and 500 inpatient beds using branded drugs would be INR 7818 (USD 174), INR 7448 (USD 166), INR 7325 (USD 163), INR 7264 (USD 161) and INR 7228 (USD 160) respectively (Figure 2).

### Fiscal Sustainability

We found that if the health care services described above were to be universalized for entire 1.2 billion Indian population, the annual cost of providing curative care will be INR 2055 billion (USD 46 billion), with a range of INR 968 billion to INR 3930 billion. The cost of preventive services has been estimated to be INR 300 per capita in India [25]. Thus, the overall cost of providing preventive and curative services will be INR 2415 billion (USD 54 billion), with a range of INR 1328 billion to INR 4290 billion. This would amount to increasing the allocation to health sector from current nearly 1% to 3.8% (2.1% to 6.8%) of GDP (Table 4).

## Discussion

Overall our study indicates that in order to universalize health care services, using generic drugs, INR 6852 (USD 152) would required to be spent per household (INR 1713 per capita per year) in India. At a per capita GDP of USD 1176 [27], this would cost India about 3.8% (2.1%–6.8%) of the GDP. Our estimates are higher than the estimate (INR 1160 per capita) of National Commission on Macroeconomics and Health (NCMH) [25]. The cost of health care has increased since the time when NCMH costs were computed. Between the two rounds of National Health Accounts in 2001–02 and 2004–05, per capita private and public health care expenditures increased by 21% and 17% respectively [28]. Rising health care expenditures in India are fuelled due to many factors related to demographic, epidemiological and social transition [25], [29], [30]. Secondly, our assumptions of staff salaries were drawn from a survey of private hospitals, whereas the NCMH sourced its salary estimates from Central Government pay scales, which are lower than private sector salaries.

We would like to note certain limitations in our analysis. Ideally, we would have preferred to use community prevalence/incidence of different diseases to estimate morbidity burden. However data on community prevalence for most diseases is scarce in India [30]. Secondly, unit cost estimates for treating different diseases is scarce in India [31], [32], [33], [34], [35]. We used the estimates available from standard treatment guidelines for selected diseases and their costing [20]. For other diseases which were not included by the consultative group, we interviewed doctors in a tertiary care hospital in Chandigarh, along with review of case records to observe the standard treatment protocols and the drugs, consumables and diagnostic investigations used. There is urgent need for developing unit cost estimates for treating different diseases. This information will also be required for estimating prospective payment schemes for providers. It is also particularly relevant since the Government of India is reimbursing providers under the *Rashtriya Swasthya Bima Yojana* (National Insurance Scheme for informal sector) on a prospectively decided payment norms. Thirdly, there is wide inter-state variation in disease burden and cost of care, whereas some of our parameters are based on one Union Territory in northern India. We accounted for variation by undertaking a sensitivity analysis to estimate the effect of parameter uncertainty on base case outcome measures. Fourthly, our analysis focused on the provision of care at secondary and tertiary level. However, we used the estimates of National Commission on Macroeconomics and Health for cost of primary health care provision in our overall estimates of cost of universalizing health care in India. Fifth, it is usually seen that demand often outstrips the estimations, when service is not charged at point of use (moral hazard at both demand and supply end). However, in order to have rational estimate of cost of care, we assumed that each episode of sickness will be reported to the hospital. This is unlike the current health seeking behavior where many minor illness episodes do not present to qualified medical practitioners, and some proportion of population (especially rich) may continue to buy private insurance with better coverage.

Beside the issue of raising the required resources for universal health care, key questions that need to be answered are: where should pooling of resources take place, how should the services be purchased and who should be providing the services? Health care in India should be largely tax-funded because, besides the argument of progressivity of general direct taxation [36], large informal sector employment precludes the option of using payroll-taxes for funding health care. Theoretical arguments and empirical evidence to a large extent does not support predominant role of private sector in universalizing health care due to its inefficiencies and inequitable nature [37], [38]. It can however continue to play a complementary or supplementary role to cover for services not covered under the ambit of universal healthcare package.

Concerns of fiscal sustainability may affect large up-scaling of public sector expenditure. However, Government of India under its Common Minimum Program had committed to raise public health expenditure to 3% of GDP [39] which was again reiterated in the 11^th^ Five Year Plan [40]. India's economy has grown at more than 6% consistently over the last 3 decades, with the rate of increase reaching 8% in last few years [1]. The tax revenues have increased from 14.9% of GDP to 16.4% of GDP which is a reflection that there is progress in terms of generation of resources for social sector spending. In case it is not possible to commit sufficient resources, to begin with, Government could consider reduction of the breadth of benefit package, i.e. proportion of people to be covered or a cost sharing mechanism such as co-payment or co-insurance. Since the latter, if not implemented correctly with necessary exclusions, can result in worsening of inequalities; targeting of services to weaker geographic areas such as rural and urban slums or for economically weaker sections such as the below poverty line population can be the starting point.

An analysis of health care spending from 1990 to 2007, shows that the State health care budgets have declined, while the Central Government's health spending has registered a consistent increase especially after the launch of National Rural Health Mission (NRHM) in 2005 [41]. Overall, it has been observed that the onus of funding universal health care lies with the Central Government [42]. Central Government should consider funding a basic healthcare package, whereas State Governments could take the onus of increasing the depth and height of the benefit package, i.e. increasing the number of conditions covered and the extent of health expenditure covered respectively.

A National Health Fund needs to be created as a statutory body which can function as a monopsonistic purchaser of health care from public and private sector, negotiating price and quality of services. However, till a time sufficient resources and managerial experience are raised, multiple risk-pools can be a starting point with a focus on equity. In the current context contributory schemes for formal sector employees may co-exist with a scheme which is funded by general taxation targeted towards below poverty line population. *Rashtriya Swasthya Bima Yojana* (RSBY), a health insurance scheme for informal sector employees which has been launched in 2008, is an example for such an arrangement of risk pool which co-exists with schemes for formal sector employees such as Employees State Insurance Scheme (ESIS) and Central Government Health Scheme (CGHS) [43]. Experience from other South Asian countries such as Thailand also suggests that this path was adopted for achieving the overall objective of universal health care [44].

One of the major challenges facing universalization of healthcare in India is shortage of staff in public sector [14], [45]. Our model does not pose any steep augmentation of human resource production. Our requirement of doctors is closer to the country's average (59 doctors per 100,000 population) [25], but slightly higher for the average of Asian countries, excluding India and China [46]. However, what merits greater attention is a more equitable distribution so that doctors are available in more remote rural areas and urban slums. In order to circumvent this health workforce crisis, purchasing of health services from both public and private sector should be considered based on standard benchmarks of quality. Emphasis should be placed on effectively involving public sector and generating a competition between the public and private providers. A recent analysis shows that public sector has the potential to reduce inequalities in access and hence needs to be promoted for universalizing health care [7]. On grounds of efficiency, we propose that the hospitals should be paid on a capitation basis, with fee payment for preventive services and those curative services which the country would like to prioritize. What we propose in our model is to pay staff salaries at par with the private sector. It can be contended that mere increase in salaries may result in staff retention, but may not necessarily translate to efficiency and quality improvement. Behavioral economists have shown that provision of incentives can be used to enhance performance [47]. Hence, we propose that the staff payments should be structured in such a way that part of payment is fixed salary, with the remainder being a performance-based payment (such as fee for service) which is linked to both quantity and quality of performance. Evidence shows that when incentives are linked to performance, the services which are monitored are performed well but the coverage and quality of services not included in performance measurement can potentially decline [48]. Hence it will require careful structuring of these performance indicators, so that it does not crowd-out any essential services so as to increase supply of services which are linked to performance indicators. The Government can play a steward role in setting up an independent performance measurement body. Performance can be measured through objective reporting of hospital on some care parameters, which are validated by independent a site inspection teams which could include community representation. This has been started in NRHM, as part of the community monitoring.

There are several challenges for delivery of quality universal health care in India. However, our analysis indicates that to universalize health care, government of India needs to commit at least about 4% of GDP for healthcare which seems to be feasible in the current economic scenario. However, several issues related to delivery strategy for ensuring quality, reducing inequities in access, and managing the growth of health care demand need to be explored.

## Supporting Information

Text S1 Technical Appendix.
(DOCX)

Figure S1 Univariate sensitivity analysis for annual household premium using generic drugs.
(TIF)
