# Supplementary material for: The Cost of Universal Health Care in India: A Model Based Estimate
Source: PLoS One. 2012 Jan 27;7(1):e30362. doi: 10.1371/journal.pone.0030362 (PMC3267714; doi:10.1371/journal.pone.0030362)
Supplement: Text S1 — Technical Appendix. (DOCX) [file pone.0030362.s001.docx]

**Technical Appendix**

***Model Overview***

We parameterized a decision model to estimate the patient burden, recurrent cost and annual premium for delivering health care to a hypothetical 100,000 population through managed care. The scope of services to be delivered through managed care at health facilities has been derived from IPHS standards. We used the community morbidity rate derived from National Sample Survey (NSS) data for India to estimate population requiring medical care on a monthly basis. The model was dependent on the probability of falling ill and seeking care to estimate number of individuals reporting to hospital. Service data for one year (2008-09) on morbidity profile from two tertiary and secondary care hospitals each in Chandigarh, India, was analyzed to derive morbidity profile (proportional morbidity rates), treatment patterns (OPD versus IPD) and average length of stay. This service data was used to derive probability distribution of different diseases in the morbid population reporting to a hospital. We collected data on standard treatment protocol, for diseases which constituted 90% of the total disease burden in a given specialty. For remainder diseases, we used a weighted average cost per illness episode for the specific specialty.

***Parameter Notations***

TP : Total Population

P : Proportion morbidity Rate

N : Sick population

P^OPD^ : Proportion patients treated on OPD (Out Patient Department) basis.

P^IPD^ : Proportion patients treated on IPD (In Patient Department) basis.

N^OPD^ : Number of patients treated on OPD basis

N^IPD^ : Number of patients treated on IPD basis

μ_j_ : Disease wise morbidity rate

^OPD^N _ij_ : Number of patients department and disease wise on OPD basis

^IPD^N_ij_ : Number of patients department and disease wise on IPD basis

^OPD^C_ij_ : Cost of treatment department and disease wise on OPD basis

^IPD^C_ij_ : Cost of treatment department and disease wise on IPD basis

^OPD^C_HR_ : Human resource cost of OPD department

^IPD^C_HR_ : Human resource cost of IPD department

C_OVD_ : Overhead costs

ω_S_ : Staff salary

C_Total_ : Total Cost

***Estimation of Specialty-wise OPD and IPD attendance***

The total number of patients expected to visit the hospital on a monthly basis derived from NSS data were distributed among different specialties. Proportional morbidity rates were used to estimate morbidity-wise OPD visits in each specialty. Ratio of patients being treated on OPD and IPD basis for each disease was multiplied by the number of OPD visits to compute estimated number of in-patients. Ratio of IPD: OPD patients obtained from the analysis of tertiary care hospital data was reduced by a factor of 3, to adjust for relatively severe nature of patients reporting a tertiary hospital.

N = TP*P .............................. (1)

N = N^OPD^ _+_ N^IPD^ ................................ (2)

P^OPD^ + P^IPD^ = 1 .............................. (3)

N = Σ ^OPD^N_ij +_ Σ ^IPD^N_ij_ .............................. (4)

Where, N_ij_ = Σ (N*μ_j_)

i = 1,2,…….k

j = 1,2,……. k

Monthly patient bed-days per morbidity were calculated by multiplying number of patients being admitted per month with average length of stay for each disease. The former was then used to estimate requirement of beds in each specialty by dividing the monthly bed-days by the number of days in a month.

***Estimation of Monthly Recurrent Cost of Patient Treatment***

Standard treatment protocols and unit cost of treatment for select conditions was obtained from a previous report. For other diseases, treatment cost per illness episode was estimated from data on treatment protocol and consumable prices estimated from a public sector tertiary hospital. It has been assumed that the cost of indoor treatment would be 1.5 times of treatment on OPD basis, since the diagnostics and treatment will be more intensive. The cost of surgeries obtained from Haryana Health Department was scaled down by 50% since this is the actual cost incurred by hospitals on respective surgeries.

***Estimation of Human Resource Requirement and Salary Expenditure***

Human resource requirement has been estimated based on the predicted quantum of work. We assumed to have one specialist each in Cardiology, Nephrology, Pulmonary Medicine, Urology, Plastic Surgery and Neonatology, based on IPHS standards. Specialist doctors (MD/ MS qualification) have been assumed to run the OPD for about 4.5 hours and examine 30 patients per day. Based on the OPD attendance per day in each specialty the number of specialist doctors in Medicine, Surgery, Gynecology, Pediatrics, Orthopedics, Psychiatry, Skin, Eye, Oral Health, ENT, Radiology and Pathology was estimated.

General Medical Officers (MBBS qualification) and staff nurses are proposed to be involved in provision of clinical care for indoor patients. One medical doctor for 8, 10 and 20 patients has been assumed for intensive, private and general medical/ surgical wards respectively. Based on the number of patients likely to be admitted each day (bed-occupancy rate), the number of general medical officers was computed. Consideration has been given to duty shifts to maintain 24-hour service delivery. In a similar manner, we assumed one staff nurse for 4, 5 and 10 patients in intensive, private and general medical/ surgical ward. Staff nurses have also been considered for assisting in operation theatres. This requirement has been estimated based on the number of surgeries likely to be conducted per day. One auxiliary nurse midwife (ANM) per 5000 population was assumed to be recruited for doing home visits.

Similarly requirement for technicians for diagnostic services and attendants for wards have been estimated in accordance to their estimated workload. One senior management official in the capacity of a chief operating officer (CEO) has been assumed to oversee the entire hospital operation along with 2 junior management (hospital administration/ public health background) consultants. We also estimated necessary support staff.

Current salary structure of different staff members obtained from a survey of four private hospitals in Chandigarh was multiplied with the number of staff members to obtain total salary expenditure for the hospital.

C_Total_ = ( Σ^OPD^C_ij_ + Σ ^IPD^C_ij_ ) + ( ^OPD^C_HR_ + ^IPD^C_HR_ ) + ( C_OVD_ ) ………… (5)

Where, C_HR_ = Σ N_ij_ * ω_S_

C_OVD =_ Σ C_L_

i = 1,2,……….k

j = 1,2,……….k

S = 1,2,………m

L = 1,2,………n
