# Supplementary figures and images for: The Cost of Universal Health Care in India: A Model Based Estimate
Source: PLoS One. 2012 Jan 27;7(1):e30362. doi: 10.1371/journal.pone.0030362 (PMC3267714; doi:10.1371/journal.pone.0030362)

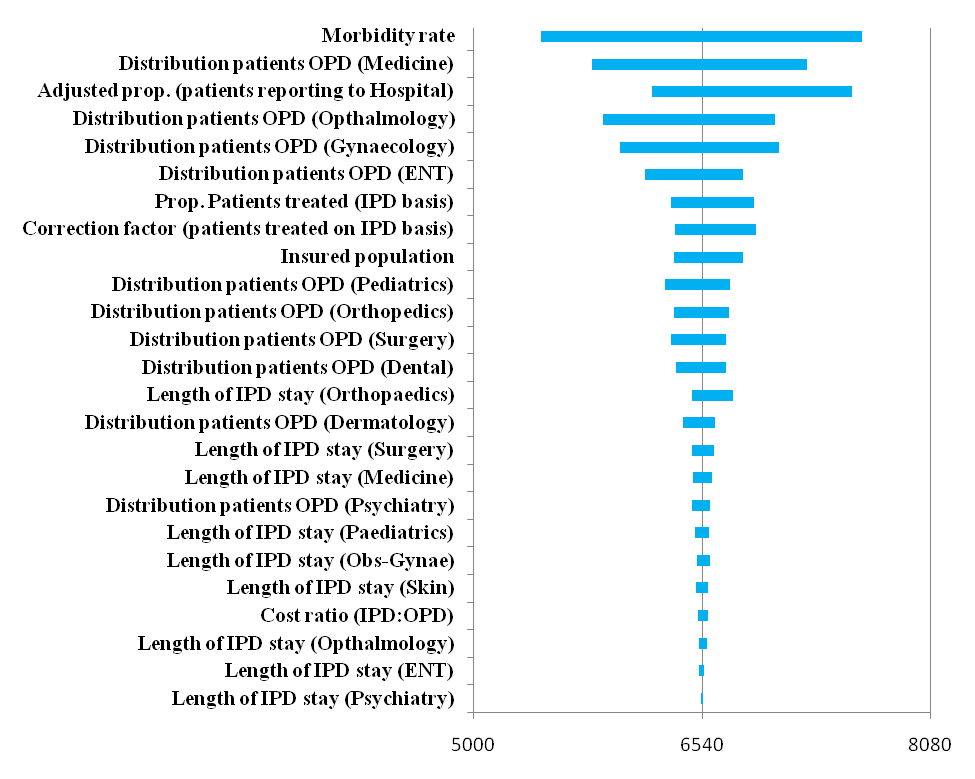

Supplement: Figure S1 — Univariate sensitivity analysis for annual household premium using generic drugs. (TIF) [file pone.0030362.s002.tif]
